# Rad51C-ATXN7 fusion gene expression in colorectal tumors

**DOI:** 10.1186/s12943-016-0527-1

**Published:** 2016-06-13

**Authors:** Arjun Kalvala, Li Gao, Brittany Aguila, Kathleen Dotts, Mohammad Rahman, Serge P. Nana-Sinkam, Xiaoping Zhou, Qi-En Wang, Joseph Amann, Gregory A. Otterson, Miguel A. Villalona-Calero, Wenrui Duan

**Affiliations:** Comprehensive Cancer Center, the Ohio State University College of Medicine and Public Health, Columbus, Ohio 43210 USA; Division of Medical Oncology Department of Internal Medicine, the Ohio State University College of Medicine and Public Health, Columbus, Ohio 43210 USA; Division of Pulmonary, Allergy, Critical Care and Sleep Medicine, the Ohio State University College of Medicine and Public Health, Columbus, Ohio 43210 USA; Department of Pathology, the Ohio State University College of Medicine and Public Health, Columbus, Ohio 43210 USA; Department of Radiology, the Ohio State University College of Medicine and Public Health, Columbus, Ohio 43210 USA; Department of Pharmacology, the Ohio State University College of Medicine and Public Health, Columbus, Ohio 43210 USA

**Keywords:** Chromosomal translocation, Rad51C, ATXN7, Fusion gene, Colorectal tumors

## Abstract

**Background:**

Fusion proteins have unique oncogenic properties and their identification can be useful either as diagnostic or therapeutic targets. Next generation sequencing data have previously shown a fusion gene formed between Rad51C and ATXN7 genes in the MCF7 breast cancer cell line. However, the existence of this fusion gene in colorectal patient tumor tissues is largely still unknown.

**Methods:**

We evaluated for the presence of Rad51C-ATXN7 fusion gene in colorectal tumors and cells by RT-PCR, PCR, Topo TA cloning, Real time PCR, immunoprecipitation and immunoblotting techniques.

**Results:**

We identified two forms of fusion mRNAs between Rad51C and ATXN7 in the colorectal tumors, including a Variant 1 (fusion transcript between Rad51C exons 1–7 and ATXN7 exons 6–13), and a Variant 2 (between Rad51C exons 1–6 and ATXN7 exons 6–13). *In silico* analysis showed that the Variant 1 produces a truncated protein, whereas the Variant 2 was predicted to produce a fusion protein with molecular weight of 110 KDa. Immunoprecipitation and Western blot analysis further showed a 110 KDa protein in colorectal tumors. 5-Azacytidine treatment of LS-174 T cells caused a 3.51-fold increase in expression of the fusion gene (Variant 2) as compared to no treatment controls evaluated by real time PCR.

**Conclusion:**

In conclusion we found a fusion gene between DNA repair gene Rad51C and neuro-cerebral ataxia Ataxin-7 gene in colorectal tumors. The in-frame fusion transcript of Variant 2 results in a fusion protein with molecular weight of 110 KDa. In addition, we found that expression of fusion gene is associated with functional impairment of Fanconi Anemia (FA) DNA repair pathway in colorectal tumors. The expression of Rad51C-ATXN7 in tumors warrants further investigation, as it suggests the potential of the fusion gene in treatment and predictive value in colorectal cancers.

**Electronic supplementary material:**

The online version of this article (doi:10.1186/s12943-016-0527-1) contains supplementary material, which is available to authorized users.

## Background

The cancer genome is characterized by mutations, deletions, amplification, chromosomal translocations and microsatellite instability. Genomic rearrangements including inversions, translocations or interstitial deletions have also been identified in malignancies. Notable examples are *TMPRSS2-ERG* in prostate cancer, and EML4-ALK fusion in non-small-cell lung tumors [1–3].

Fusion proteins have unique oncogenic properties and their identification can be useful either as diagnostic or therapeutic targets. Examples include the HHLA1-OC90 fusion transcript which is present only in teratocarcinoma cell lines [4] and the Kua-UBE2V1 fusion protein which localized to cytoplasm while UBE2V1 is a nuclear protein [5]. At the molecular level some fusion genes are formed by DNA alterations as seen with ALK-C2orf44 fusion in colorectal cancers [6]. Of therapeutic value is that inhibition of one of the genes could in some cases be enough to affect the overall activity of the fusion gene, as has been observed with oncogenic KIF5B-RET, where the cells expressing the fusion gene are sensitive to multi-kinase inhibitors which inhibit RET [7–9].

Studies using end sequence profiling and massive parallel sequencing in MCF-7 breast cancer cell lines have led to the discovery of a new fusion gene: Rad51C-ATXN7 [10]. The fusion transcript is formed between Rad51C exon (1–7) and ATXN7 (6–13). Rad51C gene resides on chromosome 17q23 and is frequently amplified in breast tumors, whereas ATXN7 is located on chromosome 3p21 [10].

Rad51C is involved in both early and late stages of homologous recombination repair, as a strand transfer protein. Rad51C and *XRCC3* (CX3)*,* and Rad51C and B (BCDX2) complexes have been shown to participate in resolution of holiday junction intermediates, but at different stages of HR [11–13]. In vitro biochemical evidence shows that the Rad51C protein forms a dimer by interaction with Rad51B*,* which exerts single stranded DNA-dependent ATPase activity [14, 15].

Moreover, defects in Rad51C have been documented as the cause of Fanconi Anemia (FA) complementation group O (FANCO) disorder [16] in which homologous recombination DNA repair in response to genotoxic insults is disrupted. Genetic and cell biological data have shown that Rad51C gene has a functional downstream role in interstrand cross links (ICL’s) during DNA repair process [16, 17]. Rad51C is shown to participate in ICL and double strand break-induced DNA damage signalling and controls intra-S-phase checkpoint through CHK2 activation [17].

The Ataxin7 (ATXN7) is one of autosomal dominant cerebellar ataxia (ADCA) which is a heterogeneous group of neurodegenerative disorders characterized by progressive degeneration of the cerebellum, brain stem and spinal cord. ADCA is caused by the expansion of the CAG repeats, producing an elongated polyglutamine tract in the corresponding protein [18]. The expanded repeats are variable in size and unstable, usually increasing in size when transmitted to successive generations [19]. This locus has been mapped to chromosome 3, and it has been determined that the diseased allele associated with spinocerebellar ataxia-7 contains 38–130 CAG repeats (near the N-terminus), compared to 7–17 in the normal allele [20]. The encoded protein is a component of the SPT3/TAF9/GCN5 acetyltransferase (STAGA) and TBP-free TAF-containing (TFTC) chromatin remodelling complexes, and it thus plays a role in transcriptional regulation [21].

The detection of the Rad51C and ATXN7 fusion gene in the MCF-7 breast cancer cell line is intriguing. However, the presence of this fusion gene in other tumor types, or in patients’ tumor specimens has not been previously documented. Here we report expression of the novel fusion gene between Rad51C and ATXN7 in human colorectal tumors, as well as association of the fusion gene with functional impairment of the FA DNA repair pathway.

## Results

### Identification of the fusion gene Rad51C-ATXN7 in colorectal tumors

**Fig. 1:**
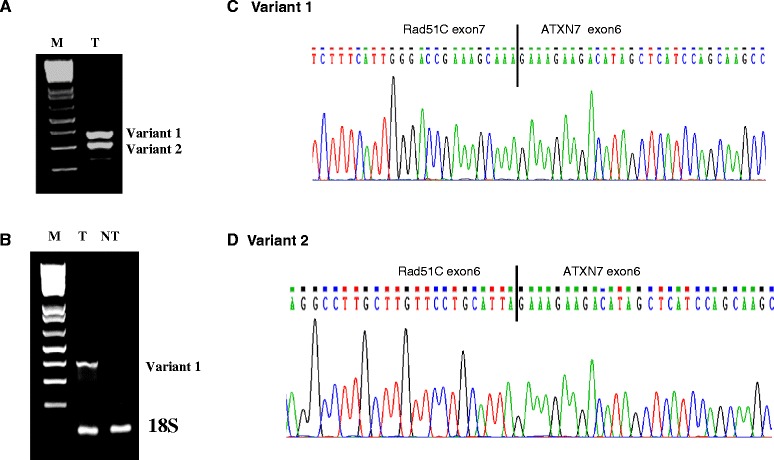
RT-PCR amplification and sequencing analysis of fusion gene Rad51C-ATXN7. Colorectal tumor (T) RNA isolated by TRIzol method, was RT-PCR amplified and sequenced. The primers (Additional file 1: Table S1) that span exon-5 of Rad51C and exon -8 of ATXN7 produced two fragments of approximately 376 bp and 316 bp (**a**). The RT-PCR amplified product from a colorectal tumor (T) showed 376 bp fragment only which was absent in the corresponding non-tumor (NT) sample (**b**). The RT-PCR amplified products were Topo TA cloned and sequenced. The RT-PCR products contained a Variant 1 of Rad51C (exon 1–7) and ATXN7 (exon 6–13) fusion gene (**c**) and a Variant 2 without exon-7 Rad51C (exon 1–6) ATXN7 (exon 6–13) (**d**). The solid bar line indicates the sequence breakpoint joining Rad51C and ATXN7

**Fig. 2:**
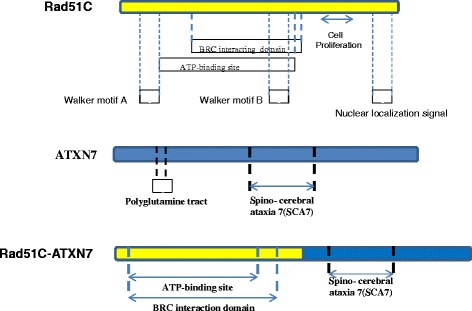
Schematic representation of portion of the fusion Variant 2 of Rad51C-ATXN7. The fusion transcript and their breakpoints are indicated with exons of Rad51C and ATXN7. The Variant 2 of the fusion gene transcript is joined at 3′-end of exon-6 of Rad51C with 5′-end of the exon-6 of ATXN7. The Fusion protein has lost its nuclear localization signal located at the C-terminus end of Rad51C and CAG (Poly glutamine tract) repeat sequence of ATXN7. The ATP binding site and BRC (BRCA1) interacting domains of Rad51C are conserved at N-terminus of the fusion protein and SCA7 (Spino- cerebral ataxia 7) domain of ATXN7 conserved at middle terminus of the fusion protein

To identify the fusion gene in colorectal tumors, Rad51C exons 1–7 (accession number NM_058216) and exons 6–13 of ATXN7 (accession number NM_000333) mRNA sequences were joined. According to the sequence, RT-PCR specific primers were designed with forward primer spanning exon-5 of Rad51C and reverse primer located in exon-8 of ATXN7. Total RNA was isolated from colorectal tumors and non-tumors following the protocol from TRIzol as mentioned in materials and methods. The cDNA containing exons 5–7 of Rad51C and exons 6-8 of ATXN7 was amplified by one step RT-PCR using primers (LF-F and LF-R; Additional file 1: Table S1) that produced either two amplicons with size of 376 bp, and 316 bp as shown in Fig. 1a, or one product with size of 376 bp (Fig. 1b). The RT-PCR products were gel purified and subjected to sequencing using the primers (LF-F and LF-R). Sequencing analysis showed that the 376 bp product, which we named Variant 1 of the fusion gene, contained Rad51C (exon 5–7) and ATXN7 (exon- 6–8) (Fig. 1c). The 316 bp fragment, named Variant 2, contained exons 5–6 of Rad51C and exons 6–8 of ATXN7 (Fig. 1d). *In silico* analysis showed that the Variant 1 extends for only three amino acids (aa) after the fusion junction, and results in a truncated protein and the Variant 2 forms an inframe fusion transcript with predicted molecular weight of protein 110 KDa (Additional file 2: Table S2). Therefore, we used the term of fusion gene to represent the Variant 2 which produces a fusion protein in the subsequent text. The structure of the gene was depicted in Fig. 2.

### Frequency of Rad51C-ATXN7 fusion gene in colorectal tumors

**Fig. 3:**
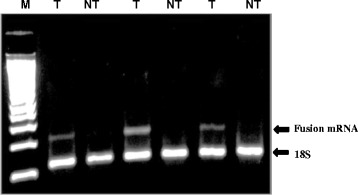
Identification and expression of the fusion gene Variant 2 Rad51C-ATXN7 by RT-PCR. A total of 67 colorectal tumor RNA (T) and their corresponding non-tumor RNA (NT) were isolated and RT-PCR amplified to identify the fusion gene. The RT-PCR amplification using primers specific for Variant 2 produces 288 bp fragments when run on 2 % agarose gel. 18 s Ribosomal rRNA was used as loading control

To identify the Rad51C-ATXN7 fusion gene in colorectal tumors, RNA was isolated from 67 tumors following TRIzol protocol. To amplify the fusion mRNA, we designed Variant specific primers according to the junction sequences between Rad51C and ATXN7 obtained previously. The primers (SJF and SJR) were listed in the Additional file 1: Table S1. Using the fusion specific RT-PCR (Fig. 3), we analysed 67 tumor samples. Of the 67 tumor RNA samples, 24 (36 %) of tumors showed the fusion mRNA (Table 1). Overall, the fusion RNA is expressed mainly in tumors (Fig. 3, Additional file 3: Table S3). We found six of the 24 tumours expressed both variants. In addition, we Topo®TA cloned 45 RT-PCR products amplified using primers LF-F and LF-R, 12(27 %) clones contained this fusion mRNA (Variant 2). In addition, Rad51C-ATXN7 fusion DNA was detected in LS-174 T cell and lung tumor genomic DNA samples by PCR analysis (Additional file 4: Figure S1).

**Table 1:** The Rad51C-ATXN7 fusion gene expression in colorectal tumors

	Tumors Tested	Fusion Gene Expression
	*N* = 67	*N* = 24 (36 %)
FANCD2 Foci negative	20 (30 %)	60 % (12/20)
FANCD2 Foci positive	47 (70 %)	26 % (12/47)
*Fishers two tailed P* Value		**0.01

^**^Indicates significant *p* value

### Rad51C-ATXN7 fusion gene expression is associated with absence of FANCD2 DNA repair foci in colorectal tumors

To assess for functional somatic deficiency in the FA repair pathway, a triple staining immunofluorescence method (FATSI) was previously developed in our lab to identify the presence of FANCD2 repair foci in the nucleus of proliferating cells [22]. Based on FATSI, 20 of 67 (30 %) colorectal tumors examined were defective for FANCD2 foci formation and 47 (70 %) were FANCD2 foci positive (Table 1). We investigated the expression of Rad51C-ATXN7 fusion gene according to FANCD2 foci status. Of the 20 FANCD2 foci negative tumors, 12 (60 %) showed expression of the fusion gene. Among the 47 FANCD2 foci positive tumors, 12 (26 %) showed expression of the fusion gene (Table 1). Thus, the fusion gene expression is more associated with foci negative tumors (*p* = 0.012*,* fisher exact two tailed test).

### Rad51C-ATXN7 and microsatellite stability

Microsatellite status was obtained from the surgical pathology report based on standard Immuno-histochemistry (IHC) analysis evaluating expression of MSH2, MSH6, MLH1 and PMS2 proteins, in the colorectal tumors [23]. Of the 67 colorectal tumors, we had 54 colorectal tumors known for microsatellite status, 43 of which were microsatellite instable (MSS) and 11 were microsatellite instable (MSI). Among the 43 MSS tumors, 13 (30 %) tumors expressed the fusion gene. Of the 11 MSI tumors, fusion gene was in 6 (55 %) tumors. The fusion gene is more expressed in the MSI tumors. However, there is no significant association statistically between the fusion gene expression and status of microsatellite (*p* = 0.166, fisher exact two tailed test).

### Effect of 5-Azacytidine on the expression of Rad51C- ATXN7 Variants

**Fig. 4:**
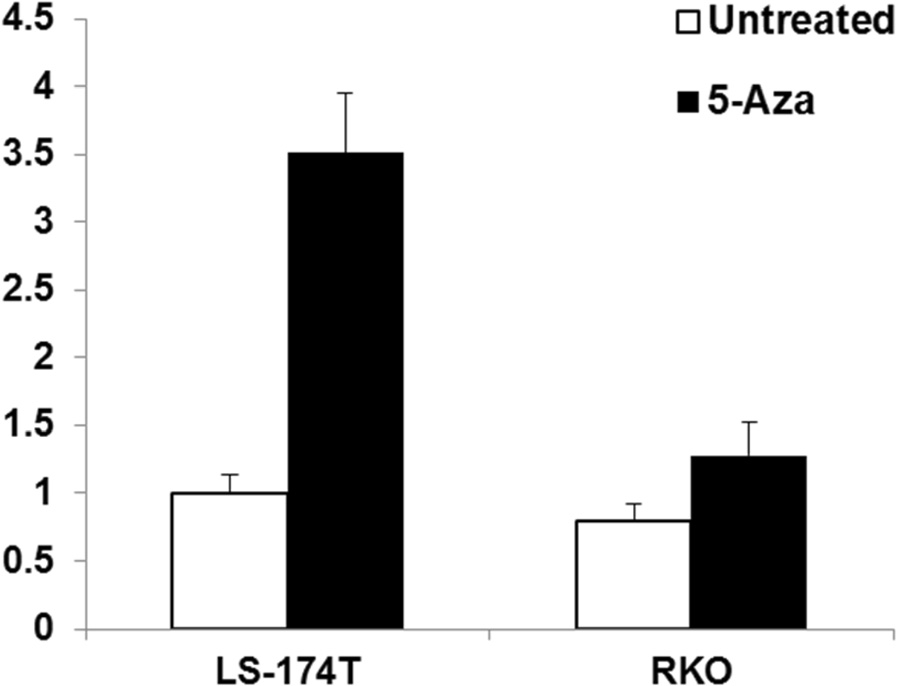
Real time PCR analysis of Rad51C-ATXN7 fusion gene Variant 2 expression in LS-174 T and RKO colorectal tumor cells pre and post 5-Azacytidine treatment. The LS-174 T and RKO cells were treated with 5-Azacytidine at dose of 5 μM for 72 h. The total RNA was isolated from pre and post 5-Azacytdine treated LS-174 T and RKO colorectal tumor cells and reverse transcribed to cDNA. The cDNA was then used as template for Rad51C-ATXN7 fusion Variant expression analysis using Taqman real time PCR. The analysis showed 3.51 fold increase in relative expression of RNA for Variant 2 in comparison to untreated control in LS-174 T cells. The RKO cells showed only 1.2 fold mild increase in relative expression of RNA for Variant 2 in comparison to untreated control

We have shown several hypermethylated CpG sites within the promoter of Rad51C [24] and treatment with hypomethylating agents causes demethylation at the three methylated CpG sites in the promoter region. We evaluated if the expression of the Rad51C-ATXN7 fusion gene could be altered by treatment with DNA hypomethylating agents. LS-174 T and RKO cells were treated with 5-Azacytidine at 5 μM for 72 h and the total RNA isolated from the cells. To quantitatively analyze the effect of promoter methylation on expression of Rad51C-ATXN7 fusion gene, RNA from 5-Azacytidine treated cells as well as pre-treatment control cells was analyzed with Taqman real time PCR. The real time PCR primers and respective amplicon sizes for Variant 2 are described in Additional file 1: Table S1. 18S ribosomal rRNA was used as endogenous control. The real time PCR analysis showed a 3.51 fold increase in the expression of the fusion gene, respectively, following treatment with 5-Azacytidine as compared to untreated controls (Fig. 4). RKO cells showed 1.2 fold mild increases in the expression of the fusion gene post treatment. Immunoprecipitation of total Rad51C protein from the post 5-Azacytdine treated LS-174 T cells showed higher expression of protein (data not shown) in comparison to the untreated control.

### Translation of fusion transcripts of Rad51C-ATXN7

The Rad51C-ATXN7 fusion transcripts identified from the colorectal tumors were *in silico* translated using EMBOSS program [25]. The fusion gene (Variant 2) produced an inframe fusion transcript with predicted molecular weight of protein 110 KDa (Additional file 2: Table S2).

**Fig. 5:**
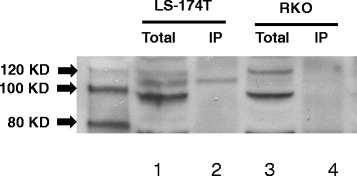
Western blot analysis of Rad51C-ATXN7 fusion protein in colon tumor cells. The immunoprecipitation pull down and immunoblot detection of the fusion protein Rad51C-ATXN7 in human colon cancer LS174T and RKO cell lines are shown. The pull down was carried out using 500 μg total cell lysates prepared from LS-174 T, RKO cells. Briefly, the total cell lysates were incubated with Dynabeads® containing protein G and 10 μg mouse monoclonal anti-Rad51C antibody. Immunoblot detection was carried out using Rabbit polyclonal anti-ATXN7 antibody and anti-rabbit secondary antibody by loading 150 μg total protein on 4–12 % NuPAGE gel. Lane 1- Total LS174T cell lysate, Lane 2- LS174T protein immunoprecipitated using anti-Rad51C antibody, Lane 3- Total RKO cell lysate, Lane 4- RKO protein immunoprecipitated using anti-Rad51C antibody. The immunoblot was detected using anti-ATXN7 antibody

To confirm the existence of the fusion protein between Rad51C and ATXN7, immunoprecipitation of Rad51C protein was carried out using Dyna MagTM -2 beads from the total cell extracts prepared from the colon cancer cell lines LS-174 T and RKO. The total protein was immunoprecipitated using anti Rad51C N-terminus antibody and the pull down proteins were analysed with Western immunoblot using anti-ATXN7 antibody. As shown in Fig. 5, a protein with molecular weight of around 110 KDa was observed in the total and Rad51C antibody immunoprecipitated samples from LS-174 T cells, which was absent in the RKO cell line.

### Differential response to the treatment of cisplatin between LS-174 T cell and RKO cell

**Fig. 6:**
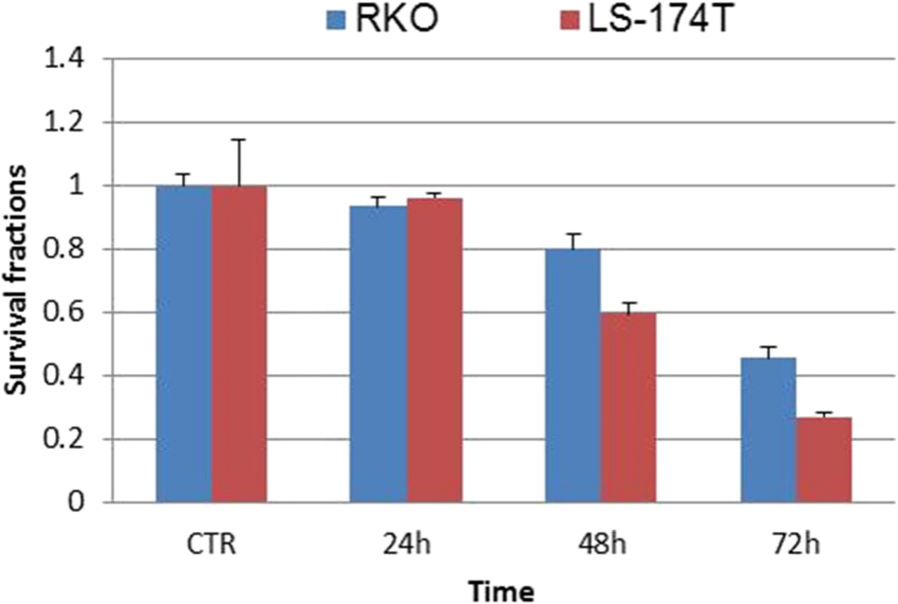
MTT assay analysis of cell survival in LS-174 T and RKO cells post treatment of cisplatin. We analysed cell survival fractions for the cells that are expressing the fusion gene (LS-174 T) as compared with non-expressing cells (RKO) post treatment of cisplatin (5 μg/ml). MTT assay was used for the cell viability analysis and an averaged absorbance was recorded 24, 48 and 72 h post treatment. Cell viability analysis showed that the LS-174 T cells had 60 and 27.5 % of viable cells compared to non-treatment controls 48 h and 72 h post treatment with cisplatin. In contrast, there were 80 and 46 % of viable cells in the RKO population 48 h and 72 h post cisplatin treatment. The LS-174 T cell was more sensitive to the treatment of cisplatin

To evaluate the influence of the fusion gene in regarding to cell viability following exposure to cisplatin, we evaluated survival fractions for the cells that are expressing the fusion gene (LS-174 T) as compared with non-expressing cells (RKO) post treatment of cisplatin (5 μg/ml). MTT assay was used for the cell viability analysis and an averaged absorbance was recorded 24, 48, and 72 h post treatment. MTT data showed that the LS-174 T cells had 60 and 27.5 % of viable cells compared to non-treatment controls 48 and 72 h post treatment with cisplatin. In contrast, there were 80 and 46 % of viable cells in the RKO population 48 and 72 h post cisplatin treatment (Fig. 6). These results indicate LS-174 T cells are more sensitive to the treatment of cisplatin, a DNA interstrand crosslinking agent.

## Discussion

Alterations in several important pathways are involved in the initiation and progression of colorectal cancers. These alterations include the common mutations in APC, TP53, SMAD4, PIK3CA and KRAS genes, and other less frequent mutations, such as in ARID1A, SOX9 and FAM123B [26, 27]. By deep sequencing (genomic or RNA sequencing) of colorectal tumors, many fusion genes have been identified [26, 27]. Further insight into these fusion genes may enable researchers to identify potential therapeutic targets.

In the current study we evaluated the frequency and expression of the fusion gene formed between Rad51C and ATXN7 in colorectal tumors and cell lines. Since Rad51C is one of the key proteins for the formation of FA homologous recombination DNA repair foci following genotoxic insults, we evaluated if the Rad51C-ATXN7 fusion protein affected FA repair foci formation.

We had previously shown a splice Variant of the Rad51C to be overexpressed in colorectal tumors [24]. Here we report the Rad51C-ATXN-7 fusion mRNA products in the same malignancy. The N-terminus domain of Rad51C has DNA dependent ATPase function, important for homologous recombination mediator activity [13, 14]. The fusion transcript Variant 1 could not produce fusion protein. However, the fusion transcript Variant 2 is able to form an inframe fusion protein which contains the N-terminus domain of Rad51C. Among 67 human colorectal tumors analyzed, there was a significant correlation between the presence of the fusion gene and lack of FANCD2 foci. Furthermore, we found that the LS-174 T cells (expressing the fusion gene) were more sensitive to the treatment of cisplatin comparing with RKO cells (non-expressing the fusion gene). These findings taken together may imply a potential role of the fusion gene in treatment and predictive value in colorectal cancers. In addition, the fusion gene is more expressed in the MSI tumors though the association between microsatellite stability and expression of fusion gene was not significant statistically.

Our results also show that the Rad51C-ATXN7 fusion gene expression was up regulated post treatment of 5-Azacytdine, a classic demethylation agent. This indicates that Rad51C promoter methylation can regulate the expression of fusion gene Variants in colorectal tumors. We have recently demonstrated several hypermethylated CpG sites in the promoter of Rad51C which is thought to regulate Rad51C isoforms [24]. Since expression of the fusion gene is regulated by treatment of 5-Azacytidine, the potential role of the fusion protein in oncogenic function or drug resistance may have clinical relevance. Functional in vitro and in vivo studies are needed to evaluate the function of the fusion gene and to assess the potential prognosis and predictive value of the Rad51C-ATXN7 fusion gene in colorectal tumors.

## Conclusion

In conclusion a chromosomal translocation between DNA repair gene Rad51C and Ataxin-7 has been identified in colorectal tumors. The in-frame fusion transcript results in a fusion protein with molecular weight of 110 KDa. In vitro 5-Azacytidine treatment of colorectal tumor cells showed expression of the fusion gene is regulated by promoter methylation. Expression of the fusion gene is correlated to FANCD2 foci deficiency in those colorectal tumors. Further investigation is needed to elucidate differential function of the Rad51C-ATXN7 fusion gene in colorectal tumors.

## Methods

### Colorectal patient tumor tissue collection

After Institutional Review Board (IRB) approval, fresh human tumor and paired non-tumor tissue samples were obtained from tissue procurement at The Ohio State University, tissues used for RNA and protein analysis were frozen in liquid nitrogen and stored in a –80 °C freezer. Tissues used for histological analysis were placed in 10 % neutral buffered formalin for 12 h, and then stored in PBS. Total RNA and DNA from the colorectal tumor tissue samples were isolated by homogenization and sequential precipitation following the protocol from TRIzol reagent (Life Technologies). RNA and DNA obtained from the colorectal tumor tissue samples was quantified at absorbance 260 nm and 280 nm using Nanodrop 2000C (Thermoscientific).

### Primer

The forward and reverse primers were designed using the Primer3 software [28]. The forward and reverse primers for the Variant 2 Rad51C-ATXN7 fusion gene are shown in Additional file 1: Table S1.

### cDNA preparation

The total RNA obtained from the colorectal cancer patients were reverse transcribed to cDNA following the Superscript™ II Reverse transcriptase protocol (Invitrogen, CA).

### PCR

The optimal temperature was determined for each pair of primers to be around 60 °C. PCR reactions were performed in 25 μl reaction volume with 1x PCR buffer, 10 mM dNTP mixture, 50 mM MgCl_2,_ 0.2 μM primer of each forward and reverse, 0.2 μl 1.0 unit Platinum ® Taq DNA polymerase following the protocol from (Invitrogen, CA). PCR conditions for each amplicon included 95 °C for 4 min, 95 °C for 30 s, annealing temperature at 60 °C for 30 s and extension at 72 °C for 30 s. Post PCR, the amplicons were analyzed on 1.5 % agarose gel.

### RT-PCR

RT-PCR was performed using one step RT-PCR Kit (Qiagen, CA). The junction specific primers for Variant 2 are SJF, SJR and the sequences are shown in the Additional file 1: Table S1, along with their respective amplicon sizes. The RT-PCR was performed in 25 μl reaction containing final concentration (1x) of 5 μl of 5x Qiagen one step RT-PCR buffer, 400 μM of each dNTPs, 0.6 μM each of forward and reverse primers, 1 μl of Qiagen One step RT-PCR enzyme mix, template RNA up to 2 μg and the volume was made up to 25 μl using PCR grade water. The PCR was carried out as reverse transcription for 34 min at 52 °C, initial PCR activation for 15 min at 95 °C, 32 cycles of initial denaturation for 30 s at 95 °C, annealing for 30 s at 60 °C, extension for 30 s at 72 °C and final extension for 4 min at 72 °C. The amplified products were gel extracted and purified using QIAquick Gel Extraction Kit (Qiagen, CA). The purified products were subjected to direct sequencing using the primers (Additional file 1: Table S1).

### Topo® TA cloning

For rapid and reliable identification of Rad51C-ATXN7 fusion gene, Topo TA cloning was performed following the protocol from (Invitrogen, CA). The obtained clones were sent for direct sequencing. To identify the Variant 2, the sequences obtained from TA clones were compared to sequences of Rad51C and ATXN7 mRNA sequences in Genbank.

### Fanconi anaemia (FA) triple staining immunofluorescence (FATSI)

The FA triple staining immunofluorescence method has been previously described [22]. Briefly, FFPE tumor tissue was cut at 4 microns, placed on positively charged slides, and stained with hematoxylin and eosin. Additional sections for immunofluorescence staining were placed in a 60 °C oven for one hour, cooled, deparaffinized, and rehydrated through xylenes and graded ethanol solutions to water in standard fashion. Antigen retrieval was performed by placing slides in Dako’s TRS antigen retrieval solution (Dako) in a calibrated vegetable steamer (Black and Decker) at 94 °C for 25 min. Slides then were placed on a Dako Autostainer for automated staining. The tissue sections were incubated with a primary antibody cocktail of rabbit polyclonal FANC-D2 antibody at a dilution of 1:1,000 and a monoclonal anti-Ki67 mouse antibody (Dako) at a dilution of 1:150, for one hour at room temperature. Sections then were incubated with a secondary antibody (FITC conjugated to anti-rabbit IgG and Alexa fluor 594 donkey anti-mouse IgG, Invitrogen) at 1:1,000 for one hour at room temperature. The sections were mounted on glass slides using a 4′ 6-diamidino-2-phenylindole (DAPI)-containing embedding medium (Vysis Dapi 1, Abbott Laboratories). The slides were analyzed under a Nikon E-400 fluorescence microscope [22].

### 5-Azacytidine (5-Aza) treatment of cells

Human colon cancer cell lines LS-174 T and RKO were purchased from ATCC® and were grown in complete Eagle’s Minimum Essential Medium with 10 % fetal bovine serum and 1 % penicillin/streptomycin at 37 °C (5 %, CO_2_). For the treatment of cells, 5-Azacytidine was filter-sterilized and added directly to the fresh cell culture media. Fresh 5-Azacytidine was added every 24 h at dose of 5 μM for 72 h. The post treatment cells were lysed, and the pellets were collected and stored at −80 °C.

### Real time PCR

The RNA expression levels of the target fusion gene were analyzed by Taqman real time PCR. The RNA was isolated from the 5-Azacytidine treated cells and reverse transcribed to cDNA using Superscript™ II Reverse Transcriptase from Invitrogen (CA, USA).

The primers for real time PCR amplification were designed using Primer3 software. The TaqMan MGB FAM labelled probes were purchased from Applied Biosystems (Foster City, CA). The 6-FAM labelled probes and the primers used for real time PCR amplification for Variant 2 are listed in Additional file 1: Table S1, along with respective amplicon sizes. The cDNA was used as template and the final reaction consisted of 1x Taqman® Fast Advanced Master Mix (Applied Biosystems, Carlsbad, CA), 3.0 μM FAM labelled probe, 10pmol/μl of forward and reverse primers, Human 18S rRNA VIC-MGB internal control, Applied Biosystems (Foster City, CA).

### Immunoprecipitation

Human colon cancer cell lines LS-174 T and RKO were grown in EMEM medium with 10 % fetal bovine serum and penicillin/streptomycin at 37 °C (5 %CO_2_). The cells were lysed in lysis buffer containing 1 mM PMSF, 0.5 mM DTT and 1 mM cocktail. The total cell lysates containing equal amounts of protein (500 μg) were immunoprecipitated using magnetic beads coupled protein G following Dynabeads® protocol (novex®, Life technologies). Briefly, total cell lysates were pre-incubated with mouse monoclonal Rad51C antibody (Santa Cruz Biotechnology, CA) overnight at 4 °C while rotating, washed and eluted the IP product in 20 μl volume using elution buffer by the supplier (novex®, Life technologies) and stored at −80 °C for detection using immunoblot.

### Immunoblot

Equal amount of proteins isolated from cells and tumor tissues were size fractionated on 4–12 % NuPAGE gel. Proteins were then transferred onto nitrocellulose membrane. The membrane was blocked with blocking buffer (5 % non fat milk). The blocked membrane was then incubated with target antibody rabbit polyclonal ATXN7 antibody (Bethyl Laboratories INC, TX) at 4 °C overnight. After washing the membrane with TBS-T (20 mM Tris, 0.9 %Nacl) for three times 20 min each, the membrane was then incubated with anti-rabbit secondary polyclonal antibody (GE Healthcare, UK) for 2 h at room temperature. The detection of specific protein binding was performed with a chemiluminescence kit (Amersham, GE Healthcare, UK).

### MTT

Five thousand cells from each line were seeded in each well of a 96-well plate 24 h before treatment. Cells were treated with cisplatin (5 μg/ml). Dimethylthiazolyl-2-5-diphenyltetrazolium bromide (MTT) dye solution (Sigma, St. Louis, MO) was added into the 96-well plate. The plate was incubated at 37 °C for 4 h, and then the treatment was terminated by adding stop solution (isopropanol with 0.04 N HCl). MTT was cleaved by live cells to a colored formazan product. Absorbance at 560 nm wavelength was recorded using a Bio-Rad microplate reader 680 (Bio-Rad Laboratories, Hercules, CA). Each treatment was repeated in triplicate. An averaged absorbance of blank values (containing all reagents except cells) was subtracted from all absorbance to yield corrected absorbance. The relative absorbance of each sample was calculated by comparing the average of corrected absorbance with an average of corrected untreated control.

## Abbreviations

5-Aza, 5-Azacytidine; ADCA, autosomal dominant cerebellar ataxia; CHK2, checkpoint kinase-2; FA, Fanconi anemia; FATSI, FA Triple staining Immunofluorescence; HR, homologous recombination; ICL, interstrand cross link; MSI, microsatellite instability; MSS, microsatellite stability

## Additional files

Additional file 1: Table S1. Primer sequences and amplicon sizes. (DOCX 15 kb) Additional file 2: Table S2.
*In silico* translation of fusion gene Rad51C-ATXN7. (DOCX 17 kb) Additional file 3: Table S3. Sybr green qPCR analysis of all 24 fusion positive tumor pairs (tumors and non-tumors). Following are the CT values for tumors (Label T1-T24), matched non-tumors (NT1-NT24) and non-template controls (C-T--). (DOCX 33 kb) Additional file 4: Figure S1. PCR amplification of Rad51C-ATXN7 fusion DNA: Genomic DNA was isolated from the LS 174 T, MCF-7, RKO, tumors (T) and non-tumors (NT). PCR was and performed using forward primer (sequence 5′-GCCCTGGGTTTTAAGGT TTT-3′) located in the intron 5 of Rad51C and reverse primer (5′ATGCATTGGCCTGGTGTT-3′) located in exon-6 of ATXN7. The expected PCR product was amplified mainly in the LS-174 T cell, human colorectal tumor (T). (PDF 91 kb)
