# Supplementary material for: Rad51C-ATXN7 fusion gene expression in colorectal tumors
Source: Mol Cancer. 2016 Jun 13;15:47. doi: 10.1186/s12943-016-0527-1 (PMC4906819; doi:10.1186/s12943-016-0527-1)
Supplement: Additional file 1: Table S1. — Primer sequences and amplicon sizes. (DOCX 15 kb) [file 12943_2016_527_MOESM1_ESM.docx]

Table S1, Primer sequences and amplicon sizes

| **Fusion gene** | **Variant type** | **Primer name** | **Primers** | **Sequence 5’ to 3’** | **Amplicon size in bp** |
| --- | --- | --- | --- | --- | --- |
| **Rad51C exon (1-7)-ATXN7 exon (6-13)** | **Both Variants** | LF-F  LF-R | PCR and sequencing Forward primer  PCR and sequencing Reverse primer | 5’-CAAATGATCAGCCTTGCAAAT-3’  5’- GGGTGATTTCAACAACTTGGA-3’ | 376 or 316 |
| **Rad51C exon (1-7)-ATXN7 exon (6-13)** | **Variant 1** | LJF  LJR | PCR Forward  PCR Reverse | 5’-GCCTAGCCCAGCAAATGAT-3’  5’-TCTTTCTTTGCTTTCGGTCC- 3’ | 180 |
| **Rad51C exon (1-6)-ATXN7 exon (6-13)** | **Variant 2** | SJF  SJR | PCR Forward  PCR Reverse | 5’-TGCATTAGAAAGAAGACATAGCTCAT-3’  5’-TCCACTTTCACAGAGGGTGTC-3’ | 288 |
| **Rad51C exon (1-6)-ATXN7 exon (6-13)** | **Variant 2** | SRF  SRR  Probe | Real time PCR Forward  Real time PCR Reverse  6 FAM | 5’-TTGATAGAAATCAGGCCTTGC-3’  5’- CCTCCTTTGCTTTTGGACAG-3’  5’-TGTTCCTGCATTAGAAAGAAGACA-3’ | 130 |
