# Supplementary material for: Rad51C-ATXN7 fusion gene expression in colorectal tumors
Source: Mol Cancer. 2016 Jun 13;15:47. doi: 10.1186/s12943-016-0527-1 (PMC4906819; doi:10.1186/s12943-016-0527-1)
Supplement: Additional file 2: Table S2. — In silico translation of fusion gene Rad51C-ATXN7. (DOCX 17 kb) [file 12943_2016_527_MOESM2_ESM.docx]

**Table S2*, In silico* translation of fusion gene Rad51C-ATXN7**

| **Coding region** | **Amino acid sequence for fusion gene Rad51C - ATXN7 Variant-2** | **Predicted**  **protein size** |
| --- | --- | --- |
| **Rad51C (exon 1-6) ATXN7(6-13)** | mrgktfrfem qrdlvsfpls pavrvklvsa gfqtaeelle vkpselskev giskaealet lqiirreclt nkpryagtse shkkctalel leqehtqgfi itfcsalddi lgggvplmktteicgapgvg ktqlcmqlav dvqipecfgg vageavfidt egsfmvdrvv dlataciqhl qliaekhkge ehrkaledft ldnilshiyy frcrdytell aqvyllpdfl sehskvrlvi vdgiafpfrh dlddlslrtr llnglaqqmi slannhrlav iltnqmttkidrnqallvpalERRHSSSSKPPLAVPPTSVFSFFPSLSKSKGGSASGSNRSSSGGVLSASSSSSKLLKSPKEKLQLRGNTRPMHPIQQSRVPHGRIMTPSVKVEKIHPKMDGTLLKSAVGPTCPATVSSLVKPGLNCPSIPKPTLPSPGQILNGKGLPAPPTLEKKPEDNSNNRKFLNKRLSEREFDPDIHCGVIDLDTKKPCTRSLTCKTHSLTQRRAVQGRRKRFDVLLAEHKNKTREKELIRHPDSQQPPQPLRDPHPAPPRTSQEPHQNPHGVIPSESKPFVASKPKPHTPSLPRPPGCPAQQGGSAPIDPPPVHESPHPPLPATEPASRLSSEEGEGDDKEESVEKLDCHYSGHHPQPASFCTFGSRQIGRGYYVFDSRWNRLRCALNLMVEKHLNAQLWKKIPPVPSTTSPISTRIPHRTNSVPTSQCGVSYLAAATVSTSPVLLSSTCISPNSKSVPAHGTTLNAQPAASGAMDPVCSMQSRQVSSSSSSPSTPSGLSSVPSSPMSRKPQKLKSSKSLRPKESSGNSTNCQNASSSTSGGSGKKRKNSSPLLVHSSSSSSSSSSSSHSMESFRKNCVAHSGPPYPSTVTSSHSIGLNCVTNKANAVNVRHDQSGRGPPTGSPAESIKRMSVMVNSSDSTLSLGPFIHQSNELPVNSHGSFSHSHTPLDKLIGKKRKCSPSSSSINNSSSKPTKVAKVPAVNNVHMKHTGTIPGAQGLMNSSLLHQPKARPStop | 110.9 Kda |
