# Supplementary material for: Rad51C-ATXN7 fusion gene expression in colorectal tumors
Source: Mol Cancer. 2016 Jun 13;15:47. doi: 10.1186/s12943-016-0527-1 (PMC4906819; doi:10.1186/s12943-016-0527-1)
Supplement: Additional file 3: Table S3. — Sybr green qPCR analysis of all 24 fusion positive tumor pairs (tumors and non-tumors). Following are the CT values for tumors (Label T1-T24), matched non-tumors (NT1-NT24) and non-template controls (C-T--). (DOCX 33 kb) [file 12943_2016_527_MOESM3_ESM.docx]

***Table S3***

**Sybr green qPCR analysis of all 24 fusion positive tumor pairs (tumors and Non-tumors). Following are the CT values for tumors (Label T1-T24), matched non-tumors (NT1-NT24) and non-template controls (C-T--) .**

| ***Tumors*** | | **Detector Name** | | | **Reporter** | **Task** | | **Ct** |
| --- | --- | --- | --- | --- | --- | --- | --- | --- |
| T1 | | SYBR1 | | | SYBR | Rad51C-ATXN7 V1 | | 26.89 |
| T1 | | SYBR1 | | | SYBR | Rad51C-ATXN7 V1 | | 26.42 |
| T1 | | SYBR1 | | | SYBR | Rad51C-ATXN7 V1 | | 26.28 |
| T2 | | SYBR1 | | | SYBR | Rad51C-ATXN7 V1 | | 26.01 |
| T2 | | SYBR1 | | | SYBR | Rad51C-ATXN7 V1 | | 26.34 |
| T2 | | SYBR1 | | | SYBR | Rad51C-ATXN7 V1 | | 26.2 |
| T3 | | SYBR1 | | | SYBR | Rad51C-ATXN7 V1 | | 27.02 |
| T3 | | SYBR1 | | | SYBR | Rad51C-ATXN7 V1 | | 27.06 |
| T3 | | SYBR1 | | | SYBR | Rad51C-ATXN7 V1 | | 27.21 |
| T4 | | SYBR1 | | | SYBR | Rad51C-ATXN7 V1 | | 27.76 |
| T4 | | SYBR1 | | | SYBR | Rad51C-ATXN7 V1 | | 27.38 |
| T4 | | SYBR1 | | | SYBR | Rad51C-ATXN7 V1 | | 27.81 |
| T5 | | SYBR1 | | | SYBR | Rad51C-ATXN7 V1 | | 27.6 |
| T5 | | SYBR1 | | | SYBR | Rad51C-ATXN7 V1 | | 27.2 |
| T5 | | SYBR1 | | | SYBR | Rad51C-ATXN7 V1 | | 27.12 |
| T6 | | SYBR1 | | | SYBR | Rad51C-ATXN7 V1 | | 28.6 |
| T7 | | SYBR1 | | | SYBR | Rad51C-ATXN7 V1 | | 28.1 |
| T7 | | SYBR1 | | | SYBR | Rad51C-ATXN7 V1 | | 28.5 |
| T7 | | SYBR1 | | | SYBR | Rad51C-ATXN7 V1 | | 27.1 |
| T8 | | SYBR1 | | | SYBR | Rad51C-ATXN7 V1 | | 27.68 |
| T8 | | SYBR1 | | | SYBR | Rad51C-ATXN7 V1 | | 27.21 |
| T8 | | SYBR1 | | | SYBR | Rad51C-ATXN7 V1 | | 28.6 |
| T9 | | SYBR1 | | | SYBR | Rad51C-ATXN7 V1 | | 28.1 |
| T9 | | SYBR1 | | | SYBR | Rad51C-ATXN7 V1 | | 28.5 |
| T9 | | SYBR1 | | | SYBR | Rad51C-ATXN7 V1 | | 28.36 |
| T10 | | SYBR1 | | | SYBR | Rad51C-ATXN7 V1 | | 28.1 |
| T10 | | SYBR1 | | | SYBR | Rad51C-ATXN7 V1 | | 28.6 |
| T10 | | SYBR1 | | | SYBR | Rad51C-ATXN7 V1 | | 27.4 |
| T11 | | SYBR1 | | | SYBR | Rad51C-ATXN7 V1 | | 27 |
| T11 | | SYBR1 | | | SYBR | Rad51C-ATXN7 V1 | | 27.2 |
| T11 | | SYBR1 | | | SYBR | Rad51C-ATXN7 V1 | | 28.07 |
| T12 | | SYBR1 | | | SYBR | Rad51C-ATXN7 V1 | | 28.12 |
| T12 | | SYBR1 | | | SYBR | Rad51C-ATXN7 V1 | | 28.76 |
| T12 | | SYBR1 | | | SYBR | Rad51C-ATXN7 V1 | | 27.51 |
| T13 | | SYBR1 | | | SYBR | Rad51C-ATXN7 V1 | | 27.38 |
| T13 | | SYBR1 | | | SYBR | Rad51C-ATXN7 V1 | | 27.81 |
| T13 | | SYBR1 | | | SYBR | Rad51C-ATXN7 V1 | | 27.98 |
| T14 | | SYBR1 | | | SYBR | Rad51C-ATXN7 V1 | | 27.15 |
| T14 | | SYBR1 | | | SYBR | Rad51C-ATXN7 V1 | | 27.12 |
| T14 | | SYBR1 | | | SYBR | Rad51C-ATXN7 V1 | | 28.96 |
| T15 | | SYBR1 | | | SYBR | Rad51C-ATXN7 V1 | | 28.12 |
| T15 | | SYBR1 | | | SYBR | Rad51C-ATXN7 V1 | | 28.96 |
| T16 | | SYBR1 | | | SYBR | Rad51C-ATXN7 V1 | | 28.91 |
| T16 | | SYBR1 | | | SYBR | Rad51C-ATXN7 V1 | | 28.76 |
| T16 | | SYBR1 | | | SYBR | Rad51C-ATXN7 V1 | | 28.91 |
| T17 | | SYBR1 | | | SYBR | Rad51C-ATXN7 V1 | | 27.76 |
| T17 | | SYBR1 | | | SYBR | Rad51C-ATXN7 V1 | | 27.89 |
| T17 | | SYBR1 | | | SYBR | Rad51C-ATXN7 V1 | | 27.13 |
| T18 | | SYBR1 | | | SYBR | Rad51C-ATXN7 V1 | | 28.22 |
| T18 | | SYBR1 | | | SYBR | Rad51C-ATXN7 V1 | | 28.06 |
| T18 | | SYBR1 | | | SYBR | Rad51C-ATXN7 V1 | | 28.91 |
| T19 | | SYBR1 | | | SYBR | Rad51C-ATXN7 V1 | | 27.76 |
| T19 | | SYBR1 | | | SYBR | Rad51C-ATXN7 V1 | | 27.90 |
| T19 | | SYBR1 | | | SYBR | Rad51C-ATXN7 V1 | | 27.88 |
| T20 | | SYBR1 | | | SYBR | Rad51C-ATXN7 V1 | | 28.52 |
| T20 | | SYBR1 | | | SYBR | Rad51C-ATXN7 V1 | | 28.96 |
| T20 | | SYBR1 | | | SYBR | Rad51C-ATXN7 V1 | | 28.12 |
| T21 | | SYBR1 | | | SYBR | Rad51C-ATXN7 V1 | | 28.12 |
| T21 | | SYBR1 | | | SYBR | Rad51C-ATXN7 V1 | | 28.96 |
| T21 | | SYBR1 | | | SYBR | Rad51C-ATXN7 V1 | | 28.76 |
| T22 | | SYBR1 | | | SYBR | Rad51C-ATXN7 V1 | | 28.45 |
| T22 | | SYBR1 | | | SYBR | Rad51C-ATXN7 V1 | | 28.34 |
| T22 | | SYBR1 | | | SYBR | Rad51C-ATXN7 V1 | | 27.69 |
| T23 | | SYBR1 | | | SYBR | Rad51C-ATXN7 V1 | | 27.38 |
| T23 | | SYBR1 | | | SYBR | Rad51C-ATXN7 V1 | | 27.07 |
| T23 | | SYBR1 | | | SYBR | Rad51C-ATXN7 V1 | | 27.71 |
| T24 | | SYBR1 | | | SYBR | Rad51C-ATXN7 V1 | | 27.19 |
| T24 | | SYBR1 | | | SYBR | Rad51C-ATXN7 V1 | | 27.84 |
| T24 | | SYBR1 | | | SYBR | Rad51C-ATXN7 V1 | | 27.57 |
|  | |  | | |  |  | |  |
|  | |  | | |  |  | |  |
|  | |  | | |  |  | |  |
| **Non Tumors** | | **Detector Name** | | | **Reporter** | **Task Ct** | |  |
|  | |  | | |  |  | |  |
| NT1 | | SYBR1 | | | SYBR | Rad51C-ATXN7 V1 | | 32.33 |
| NT1 | | SYBR1 | | | SYBR | Rad51C-ATXN7 V1 | | 32.57 |
| NT1 | | SYBR1 | | | SYBR | Rad51C-ATXN7 V1 | | 32.29 |
| NT2 | | SYBR1 | | | SYBR | Rad51C-ATXN7 V1 | | 31.91 |
| NT2 | | SYBR1 | | | SYBR | Rad51C-ATXN7 V1 | | 31.93 |
| NT2 | | SYBR1 | | | SYBR | Rad51C-ATXN7 V1 | | 31.12 |
| NT3 | | SYBR1 | | | SYBR | Rad51C-ATXN7 V1 | | 32.03 |
| NT3 | | SYBR1 | | | SYBR | Rad51C-ATXN7 V1 | | 32.67 |
| NT3 | | SYBR1 | | | SYBR | Rad51C-ATXN7 V1 | | 32.89 |
| NT4 | | SYBR1 | | | SYBR | Rad51C-ATXN7 V1 | | 31.78 |
| NT4 | | SYBR1 | | | SYBR | Rad51C-ATXN7 V1 | | 31.33 |
| NT4 | | SYBR1 | | | SYBR | Rad51C-ATXN7 V1 | | 31.22 |
| NT5 | | SYBR1 | | | SYBR | Rad51C-ATXN7 V1 | | 32.29 |
| NT5 | | SYBR1 | | | SYBR | Rad51C-ATXN7 V1 | | 32.31 |
| NT5 | | SYBR1 | | | SYBR | Rad51C-ATXN7 V1 | | 32.83 |
| NT6 | | SYBR1 | | | SYBR | Rad51C-ATXN7 V1 | | 32.12 |
| NT6 | | SYBR1 | | | SYBR | Rad51C-ATXN7 V1 | | 32.63 |
| NT6 | | SYBR1 | | | SYBR | Rad51C-ATXN7 V1 | | 32.07 |
| NT7 | | SYBR1 | | | SYBR | Rad51C-ATXN7 V1 | | 33.23 |
| NT7 | | SYBR1 | | | SYBR | Rad51C-ATXN7 V1 | | 33.77 |
| NT7 | | SYBR1 | | | SYBR | Rad51C-ATXN7 V1 | | 33.09 |
| NT8 | | SYBR1 | | | SYBR | Rad51C-ATXN7 V1 | | 33.71 |
| NT8 | | SYBR1 | | | SYBR | Rad51C-ATXN7 V1 | | 33.13 |
| NT8 | | SYBR1 | | | SYBR | Rad51C-ATXN7 V1 | | 33.22 |
| NT9 | | SYBR1 | | | SYBR | Rad51C-ATXN7 V1 | | 32.10 |
| NT9 | | SYBR1 | | | SYBR | Rad51C-ATXN7 V1 | | 32.49 |
| NT9 | | SYBR1 | | | SYBR | Rad51C-ATXN7 V1 | | 32.28 |
| NT10 | | SYBR1 | | | SYBR | Rad51C-ATXN7 V1 | | 32.03 |
| NT10 | | SYBR1 | | | SYBR | Rad51C-ATXN7 V1 | | 31.86 |
| NT10 | | SYBR1 | | | SYBR | Rad51C-ATXN7 V1 | | 31.93 |
| NT11 | | SYBR1 | | | SYBR | Rad51C-ATXN7 V1 | | 32.52 |
| NT11 | | SYBR1 | | | SYBR | Rad51C-ATXN7 V1 | | 32.58 |
| NT11 | | SYBR1 | | | SYBR | Rad51C-ATXN7 V1 | | 32.68 |
| NT12 | | SYBR1 | | | SYBR | Rad51C-ATXN7 V1 | | 32.33 |
| NT12 | | SYBR1 | | | SYBR | Rad51C-ATXN7 V1 | | 30.46 |
| NT12 | | SYBR1 | | | SYBR | Rad51C-ATXN7 V1 | | 30.09 |
| NT13 | | SYBR1 | | | SYBR | Rad51C-ATXN7 V1 | | 30.10 |
| NT13 | | SYBR1 | | | SYBR | Rad51C-ATXN7 V1 | | 31.43 |
| NT13 | | SYBR1 | | | SYBR | Rad51C-ATXN7 V1 | | 31.12 |
| NT14 | | SYBR1 | | | SYBR | Rad51C-ATXN7 V1 | | 31.23 |
| NT14 | | SYBR1 | | | SYBR | Rad51C-ATXN7 V1 | | 32.87 |
| NT14 | | SYBR1 | | | SYBR | Rad51C-ATXN7 V1 | | 32.19 |
| NT15 | | SYBR1 | | | SYBR | Rad51C-ATXN7 V1 | | 32.01 |
| NT15 | | SYBR1 | | | SYBR | Rad51C-ATXN7 V1 | | 32.99 |
| NT15 | | SYBR1 | | | SYBR | Rad51C-ATXN7 V1 | | 32.02 |
| NT16 | | SYBR1 | | | SYBR | Rad51C-ATXN7 V1 | | 32.73 |
| NT16 | | SYBR2 | | | SYBR | Rad51C-ATXN7 V1 | | 32.01 |
| NT16 | | SYBR3 | | | SYBR | Rad51C-ATXN7 V1 | | 32.73 |
| NT17 | | SYBR1 | | | SYBR | Rad51C-ATXN7 V1 | | 31.01 |
| NT17 | | SYBR1 | | | SYBR | Rad51C-ATXN7 V1 | | 31.63 |
| NT17 | | SYBR1 | | | SYBR | Rad51C-ATXN7 V1 | | 31.12 |
| NT18 | | SYBR1 | | | SYBR | Rad51C-ATXN7 V1 | | 32.83 |
| NT18 | | SYBR1 | | | SYBR | Rad51C-ATXN7 V1 | | 32.17 |
| NT18 | | SYBR1 | | | SYBR | Rad51C-ATXN7 V1 | | 32.19 |
| NT19 | | SYBR1 | | | SYBR | Rad51C-ATXN7 V1 | | 31.11 |
| NT19 | | SYBR1 | | | SYBR | Rad51C-ATXN7 V1 | | 31.03 |
| NT19 | | SYBR1 | | | SYBR | Rad51C-ATXN7 V1 | | 32.02 |
| NT20 | | SYBR1 | | | SYBR | Rad51C-ATXN7 V1 | | 32.99 |
| NT20 | | SYBR2 | | | SYBR | Rad51C-ATXN7 V1 | | 32.11 |
| NT20 | | SYBR3 | | | SYBR | Rad51C-ATXN7 V1 | | 32.93 |
| NT21 | | SYBR1 | | | SYBR | Rad51C-ATXN7 V1 | | 31.12 |
| NT21 | | SYBR1 | | | SYBR | Rad51C-ATXN7 V1 | | 32.33 |
| NT21 | | SYBR1 | | | SYBR | Rad51C-ATXN7 V1 | | 32.57 |
| NT22 | | SYBR1 | | | SYBR | Rad51C-ATXN7 V1 | | 31.29 |
| NT22 | | SYBR1 | | | SYBR | Rad51C-ATXN7 V1 | | 31.91 |
| NT22 | | SYBR1 | | | SYBR | Rad51C-ATXN7 V1 | | 31.93 |
| NT23 | | SYBR1 | | | SYBR | Rad51C-ATXN7 V1 | | 32.33 |
| NT23 | | SYBR1 | | | SYBR | Rad51C-ATXN7 V1 | | 33.47 |
| NT23 | | SYBR1 | | | SYBR | Rad51C-ATXN7 V1 | | 32.89 |
| NT24 | | SYBR1 | | | SYBR | Rad51C-ATXN7 V1 | | 31.91 |
| NT24 | | SYBR1 | | | SYBR | Rad51C-ATXN7 V1 | | 32.97 |
| NT24 | | SYBR1 | | | SYBR | Rad51C-ATXN7 V1 | | 32.32 |
|  | |  | | |  |  | |  |
|  | |  | | |  |  | |  |
| **Control** | | **Detector Name** | | | **Reporter** | **Task Ct** | |  |
|  | |  | | |  |  | |  |
|  | |  | | |  |  | |  |
| C-T1 | | SYBR1 | | | SYBR | Rad51C-ATXN7 C1 | | 35.90 |
| C-T2 | | SYBR2 | | | SYBR | Rad51C-ATXN7 C2 | | 35.67 |
| C-T3 | | SYBR3 | | | SYBR | Rad51C-ATXN7 C3 | | 34.70 |
| C-T4 | | SYBR4 | | | SYBR | Rad51C-ATXN7 C4 | | 36.95 |
| C-T5 | | SYBR5 | | | SYBR | Rad51C-ATXN7 C5 | | 34.11 |
| C-T6 | | SYBR6 | | | SYBR | Rad51C-ATXN7 C6 | | 37.54 |
|  | |  |  | | |  |  |  |
| C-T7 | | SYBR7 | SYBR | | | Rad51C-ATXN7 C7 | 37.90 |  |
| C-T8 | | SYBR8 | SYBR | | | Rad51C-ATXN7 C8 | 38.34 |  |
| C-T9 | | SYBR9 | SYBR | | | Rad51C-ATXN7 C9 | 38.90 |  |
| C-T10 | | SYBR10 | SYBR | | | Rad51C-ATXN7 C10 | 37.80 |  |
| C-T11 | | SYBR11 | SYBR | | | Rad51C-ATXN7 C11 | 38.61 |  |
| C-T12 | | SYBR12 | SYBR | | | Rad51C-ATXN7 C12 | 37.95 |  |
| C-T13 | | SYBR13 | SYBR | | | Rad51C-ATXN7 C13 | 38.09 |  |
| C-T14 | | SYBR14 | SYBR | | | Rad51C-ATXN7 C14 | 39.65 |  |
| C-T15 | | SYBR15 | SYBR | | | Rad51C-ATXN7 C15 | 37.46 |  |
| C-T16 | | SYBR16 | SYBR | | | Rad51C-ATXN7 C16 | 37.63 |  |
| C-T17 | | SYBR17 | SYBR | | | Rad51C-ATXN7 C17 | 37.04 |  |
| C-T18 | | SYBR18 | SYBR | | | Rad51C-ATXN7 C18 | 39.89 |  |
| C-T19 | | SYBR19 | SYBR | | | Rad51C-ATXN7 C19 | 38.74 |  |
| C-T20 | | SYBR20 | SYBR | | | Rad51C-ATXN7 C20 | 37.68 |  |
| C-T21 | | SYBR21 | SYBR | | | Rad51C-ATXN7 C21 | 37.09 |  |
| C-T22 | | SYBR22 | SYBR | | | Rad51C-ATXN7 C22 | 39.95 |  |
| C-T23 | | SYBR23 | SYBR | | | Rad51C-ATXN7 C23 | 38.48 |  |
| C-T24 | | SYBR24 | SYBR | | | Rad51C-ATXN7 C24 | 39.65 |  |
