# Supplementary material for: Rad51C-ATXN7 fusion gene expression in colorectal tumors
Source: Mol Cancer. 2016 Jun 13;15:47. doi: 10.1186/s12943-016-0527-1 (PMC4906819; doi:10.1186/s12943-016-0527-1)
Supplement: Additional file 4: Figure S1. — PCR amplification of Rad51C-ATXN7 fusion DNA: Genomic DNA was isolated from the LS 174 T, MCF-7, RKO, tumors (T) and non-tumors (NT). PCR was and performed using forward primer (sequence 5′-GCCCTGGGTTTTAAGGT TTT-3′) located in the intron 5 of Rad51C and reverse primer (5′ATGCATTGGCCTGGTGTT-3′) located in exon-6 of ATXN7. The expected PCR product was amplified mainly in the LS-174 T cell, human colorectal tumor (T). (PDF 91 kb) [file 12943_2016_527_MOESM4_ESM.pdf]

Supplementary figure 1

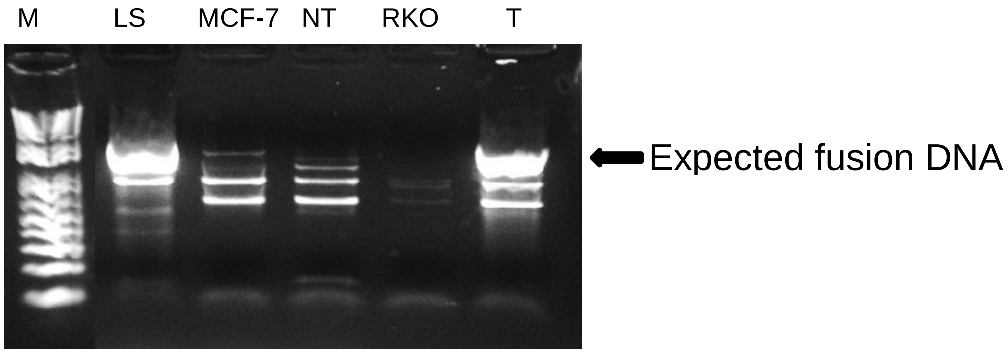

Fig. S1. PCR amplification of Rad51C-ATXN7 fusion DNA: Genomic DNA was isolated from the LS 174T, MCF-7, RKO, tumors (T) and non-tumors (NT). PCR was and performed using forward primer (sequence 5'-GCCCTGGGTTTTAAGGT TTT-3') located in the intron 5 of Rad51C and reverse primer (5'ATGCATTGGCCTGGTGTT-3') located in exon-6 of ATXN7. The expected PCR product was amplified mainly in the LS-174T cell, human colorectal tumor (T).
